# The current state of the multidisciplinary heart team approach: a systematic review

**DOI:** 10.1093/ejcts/ezae461

**Published:** 2024-12-18

**Authors:** Arian Arjomandi Rad, Sebastian Streukens, Jindra Vainer, Thanos Athanasiou, Jos Maessen, Peyman Sardari Nia

**Affiliations:** Department of Cardiothoracic Surgery, Maastricht University Medical Center, Maastricht, The Netherlands; Department of Cardiothoracic Surgery, Bristol Heart Institute, University of Bristol, Bristol, UK; Department of Cardiology, Maastricht University Medical Center, Maastricht, The Netherlands; Department of Cardiology, Maastricht University Medical Center, Maastricht, The Netherlands; Department of Surgery and Cancer, Imperial College London, London, UK; Department of Cardiothoracic Surgery, Maastricht University Medical Center, Maastricht, The Netherlands; Department of Cardiothoracic Surgery, Maastricht University Medical Center, Maastricht, The Netherlands

**Keywords:** Multidisciplinary Heart Team (HT), Cardiovascular Diseases (CVDs), Mitral Valve Interventions, Aortic Valve Interventions, Coronary Artery Disease (CAD), Standardized Outcome Metrics

## Abstract

The heart team (HT) approach, recommended for managing cardiovascular diseases, emphasizes multidisciplinary collaboration. Despite its potential benefits, evidence on its effectiveness and implementation is varied and sparse. This review assesses the HT approach’s impact on patient outcomes and care delivery in cardiovascular care. A systematic review was conducted across MEDLINE, EMBASE, PubMed, Cochrane and Google Scholar up to July 2023, focusing on studies that implemented an HT approach in coronary and heart valve disease management. Exclusion criteria included non-human studies, case reports and studies not focusing on HT outcomes. From 6270 identified articles, 20 met the inclusion criteria. These studies demonstrated significant variability in HT composition and organization, coupled with a lack of standardized metrics for evaluating clinical outcomes and the impact of the HT. Significant variability was observed in HT composition, with 13 of the 20 studies did not utilize structured templates, those that did demonstrated more consistent decision-making. In mitral valve interventions, HTs were linked to reduced in-hospital mortality and improved long-term survival (5-year survival probability of 0.74 vs 0.70, *P* = 0.04). In aortic valve interventions, 80% of patients underwent tailored valve procedures following HT evaluation. The HT approach in cardiovascular care demonstrates improved patient outcomes, particularly in specialized interventions for mitral and aortic valve diseases and coronary artery disease management. Despite these positive findings, the variability in HT implementation and the need for standardized outcome metrics call for further advances to optimize this collaborative care model.

## Graphical Abstract

**Figure:**
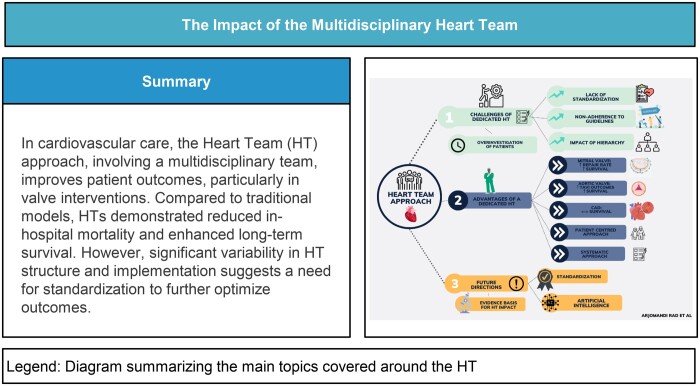

## INTRODUCTION

Cardiovascular diseases represent a global burden with increasing prevalence expected in the near future due to the population’s ageing and the steady rise of risk factors such as hypertension, diabetes and obesity [1]. Among the various treatment modalities, cardiac surgery and transcatheter interventions have been pivotal in the advancement of therapeutic outcomes. However, the complexity and diversity of cardiac pathology, coupled with the rapid evolution of diagnostic and therapeutic techniques, have created a paradigm shift in patient management—the concept of the heart team (HT) approach [2].

Traditionally, the treatment of cardiovascvular disease has been approached through the lens of individual specialities, with cardiologists primarily responsible for medical therapy and interventional procedures, and cardiac surgeons leading surgical interventions. While these specialists have made significant contributions to the field, the complex and multifaceted nature of cardiac conditions often necessitates a more collaborative and integrated approach.

The HT approach, inspired by the successful outcomes seen in other disease models such as oncology, has emerged as a novel strategy in cardiovascular care. The HT was first introduced by SYNTAX Trial [2] and later embedded as Class I recommendation by the American College of Cardiology Foundation and the American Heart Association (ACCF/AHA) in their 2012 guidelines for coronary artery disease management [3]. This approach was later extended to the management of valvular heart disease (VHD) in the 2014 ACCF/AHA guidelines [4], emphasizing the importance of a multidisciplinary team in decision-making processes for patients with severe symptomatic VHD. The European Society of Cardiology (ESC) and the European Association for Cardio-Thoracic Surgery (EACTS) have also endorsed this approach in their 2017 guidelines [5].

The HT typically comprises cardiologists, cardiac surgeons, imaging specialists and anaesthesiologists and often includes other healthcare professionals such as nurses, physiotherapists and social workers. This heterogeneity fosters a comprehensive assessment of the patient, leading to an individualized treatment plan tailored to optimize the patient’s clinical outcome and quality of life. The HT approach also facilitates the implementation of advanced therapies, such as transcatheter aortic valve implantation (TAVI) and transcatheter mitral valve repair [6]. These procedures require careful patient selection, procedural planning and post-procedural management, all of which can be effectively addressed by a multidisciplinary team.

Yet, despite these guidelines, there remains a need for strong evidence supporting the superiority of the HT approach over traditional care models in the management of cardiovascular diseases. As highlighted in our recent survey, there are wide variabilities in the conduct, format, documentation, type of patients, decision-making and auditing processes of HTs worldwide [7]. The variability in HT adoption stems from differing healthcare systems, institutional resources and professional dynamics. Factors such as team composition, meeting frequency and decision-making processes vary significantly across institutions. In some settings, HT meetings are held weekly with multidisciplinary specialists, while in others, they occur on an ad hoc basis or are inconsistently implemented. In some cases, the HT process is entirely bypassed, which highlights a significant inconsistency in adoption rates. Moreover, disparities in resource allocation, such as the availability of structured templates for decision-making or the inclusion of imaging specialists, further contribute to these inconsistencies.

Transitioning to an HT mandates a move from conventional hierarchies to a more collaborative decision-making model, requiring clear communication, consistent commitment to cross-disciplinary collaboration and a supportive institutional foundation. Additionally, scalability, particularly in settings with limited resources, remains a concern [7].

This systematic review seeks to evaluate and collate existing scientific data on the multidisciplinary HT approach’s role, effectiveness and challenges in cardiovascular care. It explores published studies on team composition, meeting formats and documentation practices and investigates the decision-making processes for inclusiveness and effectiveness. The review also examines the metrics and outcomes used to assess the HT approach’s impact. This comprehensive analysis aims to provide a nuanced and unbiased perspective, highlighting potential advantages, limitations and areas requiring further research to optimize patient outcomes and streamline care delivery.

## METHODS

### Literature search strategy

A systematic review was conducted in accordance with the Cochrane Collaboration published guidelines and the Preferred Reporting Items for Systematic Reviews and Meta-Analyses (PRISMA). MEDLINE, EMBASE, PubMed, Cochrane and Google Scholar were searched for original articles from inception to July 2023. The search terms used were as follows: (Multidisciplinary team or Interdisciplinary Team) and [cardiac or cardiology or cardiac surgery or cardiothoracic surgery or coronary revascularization or mitral valve or aortic valve or tricuspid valve or coronary artery disease or coronary artery bypass grafting (CABG) or percutaneous coronary intervention (PCI)]. Further articles were identified through the use of the ‘related articles’ function on MEDLINE and a manual search of the reference lists of articles found through the original search. The only limits used were the English language and the prespecified time frame.

### Study inclusion and exclusion criteria

All original articles published from inception to July 2023 included reporting the implementation of an HT approach for the management of coronary and heart valve disease. Studies were excluded if (i) inconsistencies in the data impeded extraction of data, (ii) the study was a review, case reports, preclinical study or abstract from a meeting, (iii) the study did not include human participants or had <10 participants, (iv) the study focused on transplantation or mechanical circulatory support, thoracic surgery, congenital cardiac surgery procedures and infective endocarditis. By following the aforementioned criteria, 3 reviewers (T.A., P.S.N. and A.A.R.) independently selected articles for further assessment following title and abstract review. Potentially eligible studies were then retrieved for full-text assessment.

### Data extraction and critical appraisal of evidence

All full texts of retrieved articles were read and reviewed by 3 authors (T.A., P.S.N. and A.A.R.) and a unanimous decision made regarding inclusion or exclusion of studies. When there was disagreement, the final decision was made by the senior author (P.S.N.), the following data were extracted: study characteristics, HT characteristics, population number, methods and main outcomes. A data extraction table for this review was developed and included the selected studies. Data extraction was performed by 2 review authors (A.A.R. and T.A.) The correctness of the tabulated data was validated by a third author (P.S.N.).

### Risk of bias analysis

**Figure 1:**
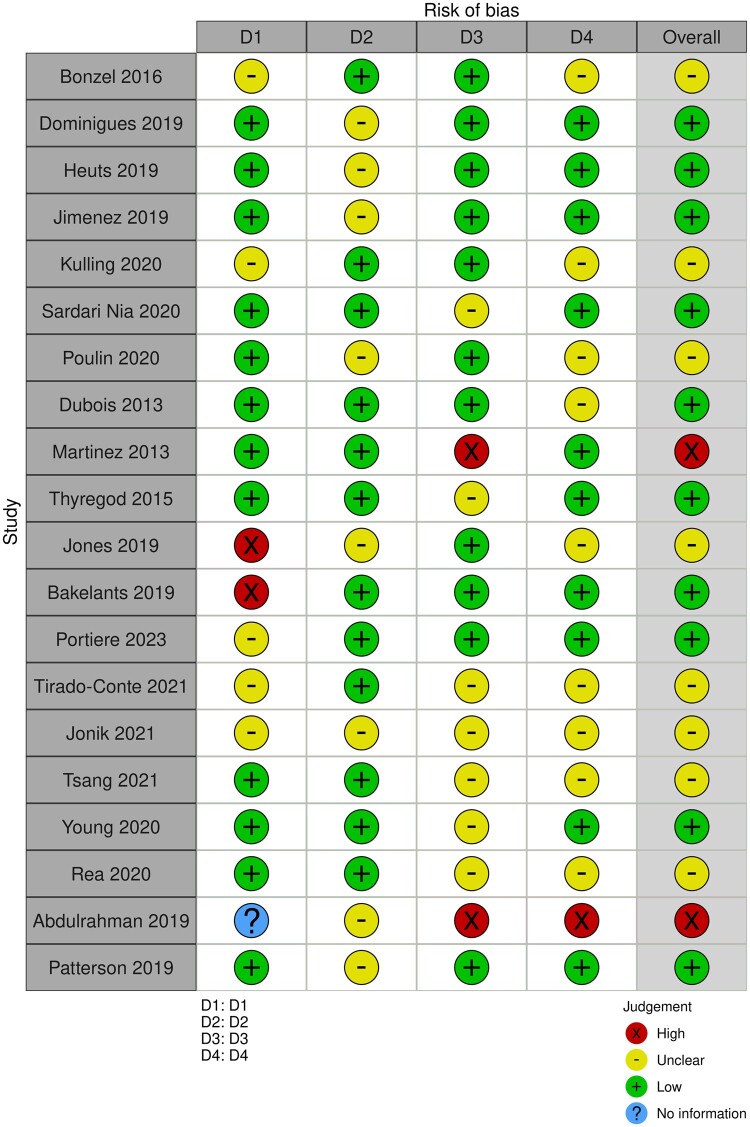
Risk of bias analysis. D1: deviation from intended intervention; D2: missing outcome data; D3: measurement of outcome; D4: selective reporting; classified as low risk of bias, some concerns or high risk of bias.

The risk of bias in the selected articles was evaluated by 2 independent reviewers (A.A.R. and T.A.) using an adapted Cochrane Collaboration risk of bias tool (Fig. 1). The methodological quality of the studies was assessed based of domains of RoB 2.0: deviation from intended intervention, missing outcome data, measurement of outcome and selective reporting, classified as low risk of bias, some concerns or high risk of bias.

## RESULTS

### Study selection

**Figure 2:**
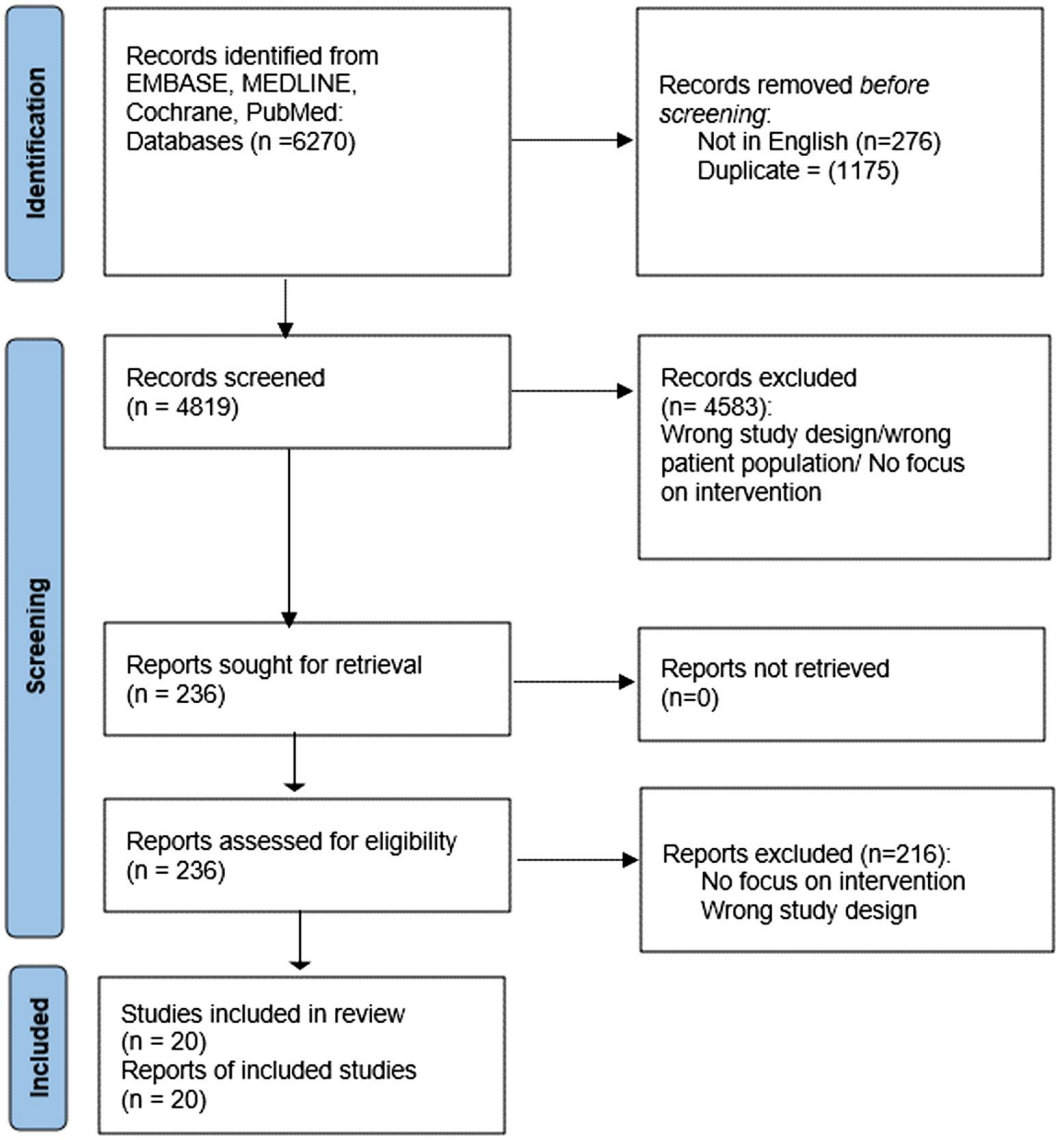
Preferred Reporting Items for Systematic Reviews and Meta-Analyses (PRISMA) Tree illustrating the systematic search and paper selection.

The literature search identified 6270 articles, of which 4819 were screened following the removal of duplicates and 236 were full-text reviewed and assessed in accordance with the inclusion and exclusion criteria. Following critical appraisal, a total of 20 studies [8–27] were included in this review. Figure 2 illustrates the entire study selection process. A summary of the studies collected and their respective designs, HT characteristics, as well as the main reported outcomes are found in Tables 1 and 2.

**Table 1:** Main characteristics of the studies focusing on CAD

Study	Year	Design	Number of patients	Study period	Area of focus	Intervention	Main findings
Bonzel *et al*.	2016	Retrospective—single centre	3408	1997–2001	All-comers adult cardiac coronary and valvular	Surgical versus transcatheter intervention versus OMT	The Heart Team approach facilitates effective clinical decisions in both ad hoc situations and planned meetings, leading to consistent clinical benefits. Evidence shows low subsequent rates of CABG and PCI following initial PCI, without an increase in mortality rates. The data’s applicability to a broad patient population underscores its universal relevance, with stable post-PCI event rates indicating a transition to stable coronary artery disease.
Domingues et al.	2018	Retrospective—single centre	1000	2010–2012	CAD	Surgical versus transcatheter intervention versus OMT	Over one-third of cases required the Heart Team to seek further diagnostic tests prior to finalizing treatment plans, with invasive cardiac imaging necessary in 29.2% of instances. Treatment advice from the Heart Team typically aligned with established clinical guidelines. The study affirmed the practicality of the Heart Team method, offering clear and accountable decision-making, with recommendations usually enacted promptly after patient referral.
Patterson *et al*.	2019	Single-centre; retrospective	245	2012–2013	CAD	Surgical versus medical versus transcatheter	Three-year follow-up indicated no significant survival difference between CABG and PCI patients, highlighting effective Heart Team decision-making. The study emphasizes the Heart Team’s crucial role in navigating complex CAD treatment choices. The Heart Team’s method integrates evidence-based medicine with diverse clinical expertise, essential for decisions in cases with clinical uncertainty.
Abdulrahman et al.	2019	Single-centre, retrospective	209	2012–2015	CAD	Surgical versus medical versus transcatheter	The study showed that the presence of senior department directors influenced Heart Team treatment decisions, favouring CABG when the surgical director was present and PCI with the cardiology director. Persistent hierarchy-based biases in Heart Team recommendations over time suggest that team composition, not just clinical guidelines, affects treatment choices. The results highlight the necessity for measures to reduce hierarchical biases to achieve more uniform, patient-focused care for multivessel CAD.
Young *et al*.	2020	Single-centre, retrospective	166	2015–2018	CAD	Surgical versus medical versus transcatheter	The Heart Team’s multidisciplinary model effectively evaluates complex coronary cases, integrating surgical and anatomical assessments. Utilizing a decision aid rooted in evidence-based management aids the Heart Team in guiding patient care. Observed in-hospital and 30-day mortality rates were 3.9% and 4.8%, respectively, within this structured team approach.
Tsang *et al*.	2020	Single centre, retrospective	245	2017–2018	CAD	Surgical versus medical versus transcatheter	The Heart Team’s treatment recommendations varied from the initial cardiologist’s in nearly a third of cases. Agreement between the Heart Team and the initial cardiologist was moderate, with a Cohen κ of 0.478. Discrepancies arose more often when considering PCI or medication over CABG. Higher discordance correlated with greater disagreement between the Heart Team’s and the original cardiologist’s interventions.

CABG = coronary artery bypass grafting; CAD = coronary artery disease; OMT = optimal medical therapy; PCI = percutaneous coronary intervention.

**Table 2:** Main characteristics of the studies focusing on valvular disease

Study	Year	Design	Number of patients	Study period	Area of focus	Intervention	Main findings
Heuts *et al*.	2019	Retrospective; single centre	158	2016–2016	MV	Surgical versus transcatheter intervention	A dedicated MV heart team achieved a 100% repair rate for degenerative MV conditions. Utilizing a specialized MV heart team for treatment decisions in MV disease proved beneficial in tailoring patient-specific therapies. While the 30-day mortality rate was higher in surgically treated patients, it improved with the involvement of a dedicated surgeon, particularly in primary, elective cases. Patients undergoing catheter-based interventions experienced higher instances of residual mitral regurgitation above grade 2 compared to those receiving surgical treatment.
Jimenez *et al*.	2019	Retrospective; single centre	103	2009–2016	MV	MV repair versus replacement	Collaborative surgical planning by a multidisciplinary valve team led to higher MV repair rates and superior repair quality in degenerative valve disease cases. The implementation of the multidisciplinary valve team approach saw MV repair rates soar from 21% to 67%. Among patients with degenerative disease, the MV repair rate reached 86.5%, with no cases necessitating reoperation. Post-repair, 95.3% of degenerative disease patients had mild or no mitral regurgitation.
Külling *et al*.	2020	Retrospective; single centre, retrospective	400	2013–2018	MV	Surgical versus transcatheter intervention	The heart team’s collaborative approach correlated with lower in-hospital mortality for MitraClip patients and elevated 4-year survival rates for those receiving surgical or percutaneous mitral regurgitation repairs. Surgical repair was reserved for low-risk patients with optimal anatomy, whereas high-risk individuals were directed to MitraClip procedures or surgical replacement. There was a notable difference in mortality risk as per EuroSCORE II across the various treatment groups managed by the heart team.
Sardari Nia *et al*.	2021	Retrospective; single centre	1145	2009–2018	MV	General Heart team versus Dedicated heart team	Referrals to a dedicated MV heart team resulted in a superior 5-year survival rate (74%) versus a general heart team (70%). The dedicated heart team’s referrals benefited from a 29% reduction in the adjusted risk of mortality compared to those managed by a general heart team. Adherence to the heart team’s recommendations significantly decreased the relative mortality risk for patients.
Poulin *et al*.	2020	Retrospective; single centre	210	2014–2018	MV	MitraClip versus patients denied MitraClip	46% of patients were ineligible for MitraClip due to various clinical reasons. After 13 months, half improved to NYHA class I or II, 63% had reduced MR severity and the mortality was 29%. The MitraClip group showed lower overall and cardiovascular mortality and less severe MR compared to matched counterparts.
Dubois *et al*.	2013	Prospective; single centre	163	2008–2011	Aortic valve	Surgical versus medical versus transcatheter	After a team evaluation, 80% of patients underwent tailored valve procedures with positive results. High-risk patients had successful TAVI procedures, matching the outcomes of lower-risk patients receiving surgical AVR Survival rates for TAVI and AVR were higher than for those on medical management alone.
Martinez *et al*.	2014	Single centre; retrospective	100	2009–2013	Aortic valve	TAVI	The heart team’s TAVI approach effectively treated high-risk patients with symptomatic aortic stenosis. TAVI showed low mortality and high success in valve implantation in the short term. Over the mid-term, TAVI maintained low reintervention rates and few major cardiac events.
Thyregod *et al*.	2016	Prospective; single centre	487	2011	Aortic valve	Surgical versus medical versus transcatheter	The heart team’s process ensured a high intervention rate, with only 7% of severe aortic stenosis patients not receiving an intervention. TAVI recipients were typically older with histories of CABG, obesity and COPD Patients on medical therapy alone had poor survival rates, with only 57.1% and 25.7% living past 1 and 3 years, respectively.
Jones *et al*.	2018	Retrospective; multicentre	3399	2006–2016	Aortic valve	Impact of the introduction of the heart team	A TAVI programme correlated with reduced mortality in severe AS patients, regardless of broader intervention access. Post-TAVI era patients were older with worse AS and ejection fraction, but had fewer comorbidities. Even after accounting for valve interventions, a significant survival benefit remained (HR 0.84).
Bakelants *et al*.	2019	Prospective; single centre	405	2008–2015	Aortic valve	Surgical versus medical versus transcatheter	A multidisciplinary heart team effectively manages severe AS in high-risk patients through successful aortic valve implantation. TAVI serves as a viable option for patients ineligible for surgical replacement, ensuring safety and efficacy. Survival rates for patients treated with TAVI or surgical replacement surpass those on medical management alone.
Rea *et al*.	2020	Multicentre, retrospective	243	2011–2020	Aortic valve	Surgical versus medical versus transcatheter	Over a 5-year period, 243 patients were evaluated for heart treatments at MHT meetings, with most recommended for TAVI, showing no significant age or EuroSCORE II differences across treatment groups. The number of patients discussed increased over time, without age changes, but with a significant decrease in mean EuroSCORE II. Survival rates 1 and 2 years post-TAVI and SAVR were comparable to those of the age-matched general population and superior to those receiving medical therapy.
Jonik *et al*.	2021	Single centre; retrospective	482	2016–2019	Aortic valve	Surgical versus medical versus transcatheter	The study underscores the Heart Team’s crucial role in guiding treatment for aortic stenosis, endorsing interventional methods for enhanced patient results. Severe AS patients experienced improved outcomes and life quality post-TAVI or SAVR, outperforming OMT alone. Both TAVI and SAVR showed superiority over OMT in key outcomes like mortality, stroke incidence and rehospitalization rates. Quality of life assessments pre- and post-Heart Team evaluations revealed no significant differences between TAVI and SAVR patient groups.
Tirado-Conte *et al*.	2021	Prospective, single centre	286	2014–2017	Aortic valve	Surgical versus medical versus transcatheter	The Heart Team was instrumental in the decision-making for aortic stenosis patients, ensuring thorough and informed evaluations. Utilization of a local consensus document alongside clinical practice guidelines helped in pinpointing candidates for Heart Team deliberation. Decision-making by the Heart Team was significantly influenced by 6 key factors: patient age, logistic EuroSCORE, presence of significant MR, patient frailty, estimated glomerular filtration rate and the STS score.
Porterie *et al*.	2023	Retrospective; single centre	528	2007–2016	Aortic valve	Heart Team versus non-heart team	The matched group managed by the Heart Team exhibited a notably lower in-hospital mortality rate (0% compared to 6.0%). Patients in the Heart Team group experienced significantly fewer complications, including stroke, low cardiac output states, reexploration for bleeding, pneumonia and extended periods of ventilation. Long-term survival and readmissions due to cardiovascular issues were comparable between patients managed by the Heart Team and those who were not.

AS = aortic stenosis; CABG = coronary artery bypass grafting; HR = hazard ratio; MR = mitral regurgitation; MV = mitral valve; NYHA = New York Heart Association; OMT = optimal medical therapy; STS = Society of Thoracic Surgeons; TAVI = transcatheter aortic valve implantation.

### Heart team structure

HT structures and details about their design are found in Tables 3 and 4.

**Table 3:** Studies focusing on CAD, structure and composition of the HT

Study	Year	Pathology	Cases discussed	Team composition	Meeting frequency	Presence of a structured template for discussion	Risk scores considered	Case review process
Bonzel *et al*.	2016	All adult cardiac coronary and valvular disease	All comers	At least 1 interventional cardiologist, 1 cardiac surgeon and 1 non-interventional cardiologist	Weekly +-emergency meeting	No mention of structured template	Yes—SYNTAX and STS	Patients received comprehensive clinical and catheterization data presentations Presentations included large-screen projections of still images and cine-angiograms
Domingues *et al*.	2018	CAD	All-comers CAD	A cardiothoracic surgeon, a clinical cardiologist and an interventional cardiologist	Daily	Yes—structured template for patient discussion	Yes—SYNTAX score	The Heart Team evaluates patient details and risk assessments provided in institutional briefings They analyse coronary images and compute SYNTAX scores for intricate CAD cases during their meetings
Patterson *et al*.	2019	CAD	CAD patients >18 years old	At least 1 interventional cardiologist, 1 cardiothoracic surgeon and 1 non-invasive cardiologist	Once a week	No mention of template. HT coordinator present	SYNTAX score	The Heart Team’s treatment decisions were informed by comprehensive clinical data, patient functionality and characteristics, with open, evidence-based discussions shaping the choice of treatment Risk evaluations included SYNTAX score calculations, and left ventricular dysfunction was classified by an ejection fraction under 50%
Abdulrahman *et al*.	2019	CAD	Isolated multivessel CAD	At least 1 interventional cardiologist, 1 cardiothoracic surgeon and 1 non-invasive cardiologist	Once a day (during weekdays)	Yes—Proforma mentioned	Yes—SYNTAX and EuroSCORE II considered	For patients with single or 2-vessel disease undergoing diagnostic coronary angiography, immediate stent placement is common, but significant left main or 3-vessel disease prompts discussion at the day’s later HT meeting An HT proforma is prepped before these meetings to compile patient demographics, comorbidities, angiographic findings and pertinent scores, ensuring efficient and informed HT discussions
Tsang *et al*.	2020	CAD	Multivessel CAD	One interventional cardiologist, 1 cardiovascular surgeon and 1 non-invasive cardiologist	N/A	Yes—structured online case presentation and a virtual heart team interface	EuroSCORE II, SYNTAX score, STS score	Eight trios of heart team members assessed patient cases via a virtual platform, making independent, blind treatment recommendations These recommendations were then contrasted with the original decisions of the treating interventional cardiologists
Young *et al*.	2020	CAD	Complex CAD and deemed high risk for surgical or percutaneous revascularization	One referring team physician, 1 primary cardiologist, at least 2 interventional cardiologists and at least 2 cardiothoracic surgeons	Upon request by referring cardiology	Yes—“CAD Heart Team Decision Aid”	STS-PROM, SYNTAX score	Patient evaluations encompassed lab results, coronary angiography, echocardiography, potential right heart haemodynamic and when necessary, non-invasive functional tests, alongside a thorough risk assessment using established tools

CAD = coronary artery disease; AVR = Aortic Valve Replacement; COPD = Chronic Obstructive Pulmonary Disease; MHT = Multidisciplinary Heart Team; TAVI = Transcatheter Aortic Valve Implantation; SAVR = Surgical Aortic Valve Replacement.

**Table 4:** Studies on focusing on valvular disease, structure and composition of the HT

Study	Year	Pathology	Cases discussed	Team composition	Meeting frequency	Presence of a structured template for discussion	Risk Scores considered	Case review process
Heuts *et al*.	2019	MV disease	Any isolated or concomitant MV pathology	One MV surgeon, 1 interventional cardiologist experienced in catheter-based MV therapies and 2 imaging cardiologists	Once a week and took place only if all members were present	Yes—Electronic custom-made heart team form integrating EU guidelines	Yes—EuroSCORE II and NYHA classification	Every patient undergoes a TTE to assess the severity and mechanism of MV disease. Should late complications or MR recurrence arise, patients are re-evaluated by the MV heart team.
Jimenez *et al*.	2019	MV disease	Adult patients referred for a MV operation	Faculty from anaesthesia, cardiology and cardiac surgery. At least 1, but up to 3, cardiologists board-certified in cardiac echocardiography were present	Once a week	No mention of template	NYHA Classification	Patients with severe MR were evaluated for repair, pinpointing the specific cause of regurgitation. TEE was utilized to identify the anatomical mechanism of MR in degenerative cases, guiding the formulation of a surgical repair plan.
Külling *et al*.	2020	MV disease	Adult patients treated for MR	Two MV surgeons (each with more than 600 MV repairs); 3 interventional cardiologists (the main operator with more than 500 mitral cases), imaging specialists and cardiac anaesthetists	Once a week	No mention of template	EuroSCORE II	Anatomical suitability for MV repair was assessed through detailed imaging using 3-dimensional transoesophageal echocardiography.
Poulin *et al*.	2020	MV diseased	Patients with moderate to severe MR were referred to the transcatheter MV program	Three interventional echocardiographers, 2 heart failure specialists, 2 cardiothoracic surgeons and 2 interventional cardiologists. Electrophysiologists were consulted as needed	Once every 2 weeks	No mention of template	STS-PROM; NYHA	Patients underwent comprehensive local echocardiographic assessments, both transthoracic and transesophageal, with additional evaluations conducted as necessary. Each patient received thorough ischaemic stratification through either invasive or non-invasive methods to inform treatment planning.
Sardari Nia	2021	MV disease	Any isolated or concomitant MV pathology	MV surgeons (performing more than 25 MV procedures per year), interventional cardiologists specialized in catheter-based MV interventions and imaging cardiologists with expertise in advanced echocardiography and MV pathology	Once a week and took place only if all members were present	Yes—Electronic custom-made heart team form integrating EU guidelines	EuroSCORE II and NYHA classification	Referred patients initially received transthoracic echocardiography at the referring site. For a conclusive treatment decision, the dedicated heart team might request further evaluations or additional tests at the MV outpatient clinic.
Dubois *et al*.	2013	Aortic valve disease	Symptomatic adults with severe AS	Three interventional cardiologists, 2 cardiac surgeons and 3 non-invasive imaging and clinical cardiologists. For specific cases, advice from geriatricians was also considered by the heart team	Once a week	No mention of template	STS Score, EuroSCORE II	Patients received a comprehensive workup encompassing clinical assessment, lab tests and in-depth imaging. Functional status was gauged using an activities of daily living index, cognitive screening and a 6-min walk test.
Martinez *et al*.	2014	Aortic valve disease	Patients with AS selected for TAVI	Three interventional cardiologists, 2 cardiac surgeons and 3 non-invasive imaging and clinical cardiologists. In particular cases, the heart team also sought input from geriatricians	N/A	No mention of a template	EuroSCORE II	Preprocedure assessments for patients include detailed echocardiography, coronary and peripheral angiography, CT scans of the heart and aorta, spirometry and carotid ultrasound.
Thyregod *et al*.	2016	Aortic valve disease	Adult patients with severe aortic stenosis	One experienced cardiac surgeon and 3 cardiologists, including the primary cardiologist, an imaging specialist and an interventional cardiologist	Daily	No mention of a form	EuroSCORE II and STS-PROM score	Patients over 18 years with severe aortic valve stenosis were consecutively referred for intervention and assessed daily by the Heart Team.
Jones *et al*.	2018	Aortic valve disease	Adult patients with severe AS	One or 2 operating structural heart specialists trained in TAVI, 1 or 2 cardiothoracic surgeons, 1 or 2 non-interventional cardiologists specializing in HF imaging, or cardiogeriatrics, 1 or 2 vascular surgeons, a radiologist specializing in structural cardiac imaging, 1 or 2 cardiac anaesthetists, fellows and nursing staff	Twice a week	No mention of a template	No risk scores included	All TAVI cases and complex SAVR procedures are considered, with a thorough review of patient history, comorbidities and diagnostic investigations.
Bakelants *et al*.	2019	Aortic valve disease	Severe symptomatic AS	Interventional cardiologists, cardiac surgeons, non-invasive imaging and clinical cardiologists	Once a week	No mention of a template	EuroSCORE II, STS Prom	Patients received a comprehensive workup: clinical and lab evaluations, imaging (echocardiography, catheterization, CT) and functional assessments (daily living activities index, cognitive testing and walking capacity).
Rea *et al*.	2020	Aortic valve disease	All patients considered too high risk of SAVR	cardiologists, cardiac surgeons, anaesthetists, cardiovascular intensivists, geriatricians and TAVI nurse coordinator	Every 2 weeks to once a month	No mention of template	EuroSCORE II	Baseline and procedural data were sourced from clinical records and MHT meeting minutes, with EuroSCORE II calculated for each patient.
Tirado-conte *et al*.	2021	Aortic valve disease	All patients with AS referred TAVI or with undecided treatment plan	At least 2 cardiac surgeons, 2 interventional cardiologists, 1 imaging cardiologist and 1 clinical cardiologist	Once a week	Yes—structured template present including risk stratification	STS-PROM, EuroSCORE II	The Heart Team identified potential TAVI candidates or those with undecided management using a local consensus document aligned with clinical practice guidelines for discussion in their meetings.
Jonik *et al*.	2021	Aortic Valve Disease	All comers with severe AS	interventional cardiologists, cardiac surgeons, clinical cardiologists and non-invasive imaging specialists	Once a week	No mention of template	EuroSCORE II, STS score	Patients across all groups were evaluated using echocardiography, with the OMT- group assessed at the time of Heart Team discussion and the SAVR- and TAVI-groups before and after intervention. The Heart Team deliberated on each patient’s clinical data, angiographic and echocardiographic results, risk factors, long-term prognosis and personal treatment preferences.
Porterie *et al*.	2023	Aortic valve disease	Octagenarians symptomatic AS	3 cardiac surgeons. 3 interventional cardiologists. 1 non-invasive cardiologist. 1 radiologist. 2 nurse practitioners.	Once a week	No mention of template	EuroSCORE II	Patients underwent comprehensive multidisciplinary clinical screening and a range of imaging modalities, including echocardiography (both transthoracic and transesophageal), multislice computed tomography and coronary angiography. This thorough evaluation ensured a complete understanding of each patient’s condition to inform treatment planning.

AS = aortic stenosis; CT = computed tomography; MR = mitral regurgitation; MV = mitral valve; NYHA = New York Heart Association; OMT = optimal medical therapy; TAVI = Transcatheter Aortic Valve Implantation; TTE = Transthoracic Echocardiography; SAVR = Surgical Aortic Valve Replacement.

- *Composition*: A total of 16 studies specified the exact number of specialty doctors involved in cardiac care. Of these, 6 studies reported the presence of more than 2 cardiologists and cardiothoracic surgeons. Eight specifically reported the inclusion of other specialties outside of cardiology and cardiothoracic/cardiac surgery. These specialties varied and included anaesthesiology, echocardiography, heart failure specialists, electrophysiologists, imaging specialists, geriatricians, clinical nurse consultants, respiratory physicians, radiologists, cardiac anaesthetists, vascular surgeons, cardiovascular intensivists and TAVI nurse coordinators. One study by Sardari Nia *et al*. proposed directly involving patients in the HT meeting.
- *Meeting frequency*: The data from the 20 studies reveal that the meeting frequency of HTs is predominantly weekly, with 11 out of the 20 studies reporting a weekly schedule. Daily meetings are reported in 3 studies, biweekly to monthly meetings are held as per 2 studies, and 1 study mentions meetings upon request. The remaining 3 studies did not specify the meeting frequency.
- *Structured discussions*: The use of structured templates and risk scores in HT meetings varies among the studies. Structured templates for patient discussion are explicitly mentioned including custom-made forms, electronic forms integrating guidelines, proformas, structured online case presentations, a ‘CAD Heart Team Decision Aid’ and templates that include risk stratification. Surprisingly, in our review, a majority of studies (13 out of 20) did not specify the use of such structured templates. Risk scores were commonly used for patient assessment, particularly the SYNTAX score in 5 studies, EuroSCORE II in 9 studies, STS-PROM in 4 studies and New York Heart Association (NYHA) classification in 3 studies.

### Impact of the heart team on clinical outcomes

Among all 20 studies [8–27], a great heterogeneity and variability were present in the reported outcomes, with a lack of consistency in the objective and methodology of the studies attempting to assess the impact of the HT approach on clinical outcomes.

#### Mitral valve team

In our systematic review, 5 studies delved into the effects of the HT on mitral valve-related outcomes. Heuts *et al.* [10] highlighted a dedicated approach that achieved a 100% repair rate for degenerative mitral valves. Collaborative surgical planning with a multidisciplinary team led to enhanced repair rates, especially for degenerative valve disease patients. Külling *et al.* [12] linked the team approach to reduced in-hospital mortality rates for MitraClip procedures and observed high 4-year survival rates for various repair methods. Sardari Nia *et al.* [13] showed that dedicated mitral valve HT referrals led to a higher 5-year survival probability compared to general HT referrals.

#### Aortic valve

Our systematic review highlighted 9 key studies examining the HT’s role in aortic valve interventions. Dubois *et al.* [15] reported that after HT evaluations, 80% of patients underwent tailored valve procedures with positive outcomes. Bonzel *et al.* [8] highlighted that only a small portion of patients with severe degenerative aortic valve stenosis were not recommended an intervention, underscoring the HT’s thorough evaluation. Thyregod *et al.* [17] observed a high intervention rate with TAVI recipients typically being older with more complex health histories. Jones *et al.* [18] found that the introduction of TAVI programs correlated with reduced mortality. Jonik *et al.* [22] reported improved outcomes and quality of life post-TAVI or SAVR (Surgical Aortic Valve Replacement), significantly outperforming optimal medical therapy alone. Porterie *et al.* [20] reported lower in-hospital mortality and fewer complications in patients managed by the HT. The HT group had significantly lower in-hospital mortality, reduced risks of complications and demonstrated equivalent long-term survival rates and readmissions for cardiovascular reasons.

#### Coronary artery disease

In our systematic review, 6 studies illuminated the HT’s pivotal role in coronary artery disease (CAD) interventions. Domingues *et al.* [9] highlighted the detailed approach of the HT, noting that additional investigations were requested in 29.2% of cases prior to making definitive treatment recommendations. Patterson *et al.* [27] delved into survival rates, finding no significant difference in outcomes for patients undergoing CABG versus those who chose PCI post-HT discussions, with a 3-year survival rate mentioned. Abdulrahman *et al.* offered insights into the decision-making dynamics, revealing that choices between CABG or PCI often mirrored the existing hierarchy within the team. The potential of seamlessly integrating a multidisciplinary HT into standard institutional practice, especially for challenging CAD cases, was articulated by Young *et al.* [24] A particularly revealing observation came from Tsang *et al.* [23], where the HT’s treatment recommendations diverged from the initial suggestions of interventional cardiologists in 30.3% of the cases.

### Contrasting general heart team and dedicated heart team

In the study by Sardari Nia *et al.* [13], patients referred to a dedicated mitral valve HT were contrasted with those referred to a general HT. Notably, the dedicated HT patients demonstrated superior 5-year survival probabilities and diminished adjusted relative risk of mortality. Adhering to the HT’s recommendations further curtailed the relative risk of mortality. The study by Jones *et al.* [18] assessed the influence of a TAVI-dedicated program’s introduction. It was observed that a TAVI program was associated with a mortality benefit for patients with severe aortic stenosis, irrespective of the broadened access to intervention. This mortality benefit was maintained even after accounting for the implementation of aortic valve intervention.

## DISCUSSION

**Figure 3:**
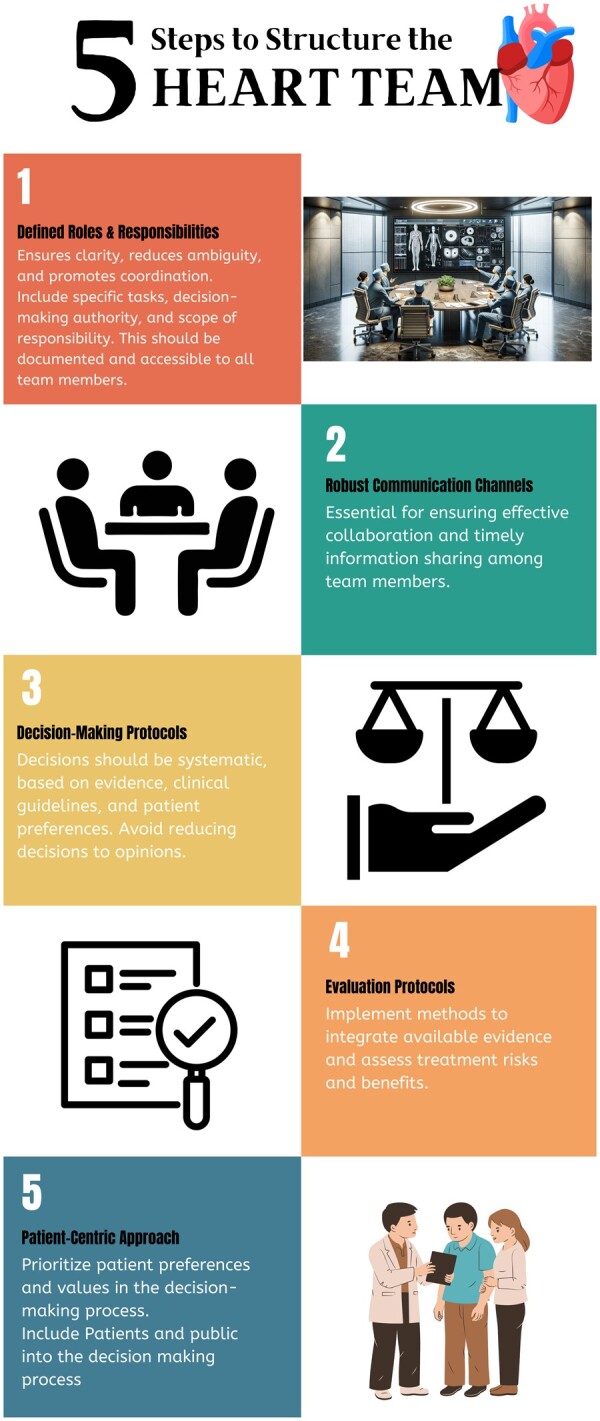
Five key steps on how to provide a structured heart team approach.

**Figure 4:**
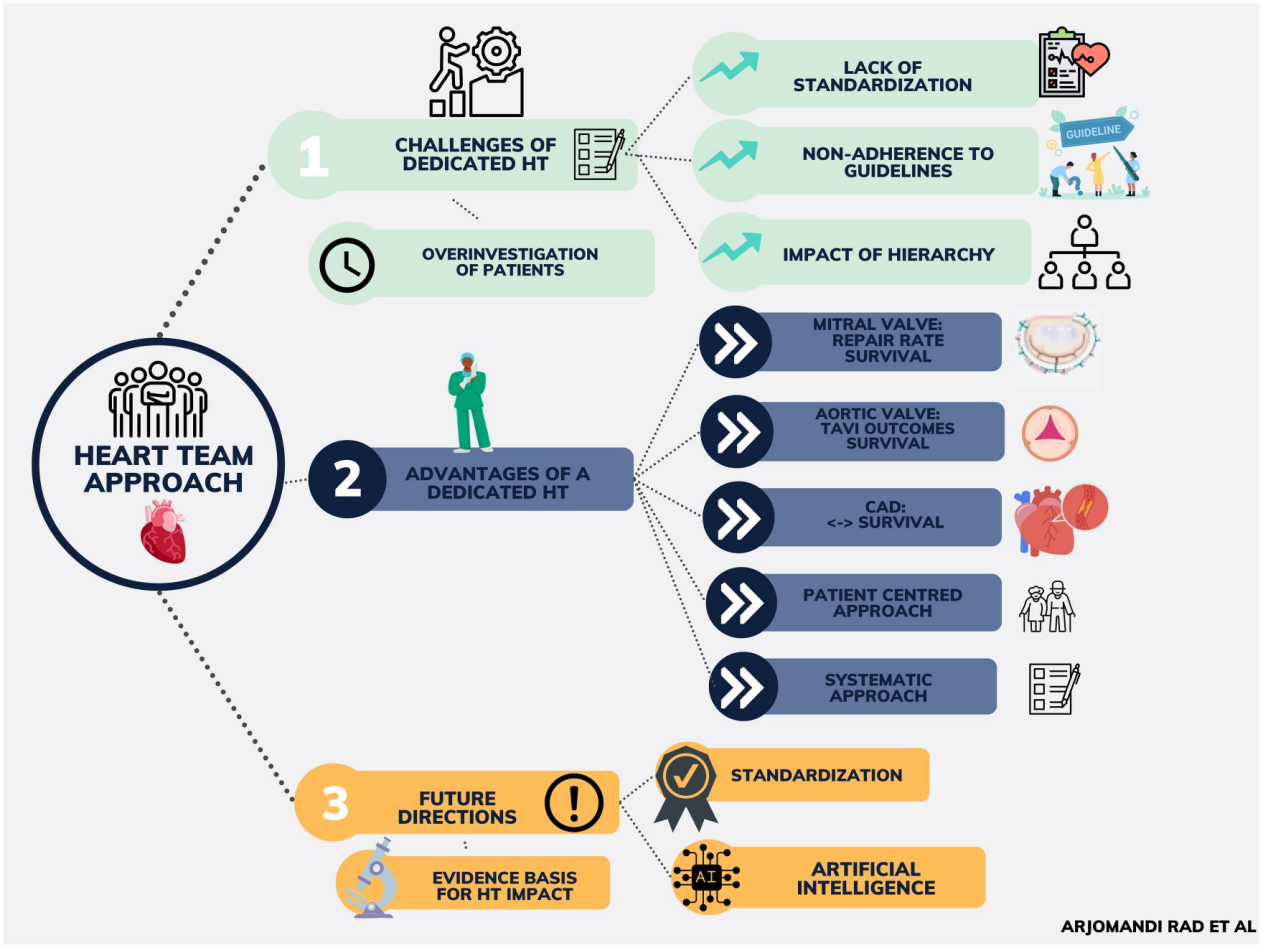
Illustration of the main topics surrounding the heart team discussed in the manuscript.

Our systematic review aligns with previous survey findings, highlighting a persistent lack of structured implementation of the HT concept in managing cardiovascular disease (Figs 3 and 4). Despite widespread acknowledgement of its importance for complex patient care, significant variability exists in team composition, meeting conduct and decision-making processes across institutions. While the overall quality of the evidence remains heterogeneous, the systematic review data highlight the effectiveness of dedicated HTs in both CAD, mitral and aortic valve disease management, with significant improvements in repair rates, survival probabilities and reduced mortality risks. Nevertheless, the lack in consistency of reported outcomes remains an important shortfall of this approach. While the HT model has been widely acknowledged as essential for managing complex cases, variability in its implementation is influenced by the resources, manpower and financial constraints of individual institutions. Each HT must adapt to the unique context of its healthcare setting, ensuring that decisions are ultimately centred around patient care rather than institutional capabilities.

### The structure and functioning of the heart team based on the literature

Our systematic review has revealed a significant discrepancy between the theoretical framework of the HT concept and its practical application. Despite clear recommendations from scientific societies such as the American College of Cardiology (ACC), the AHA, the ESC and EACTS regarding the use of a collaborative HT approach as a class I indication in the management of cardiovascular disease [3–5], our findings indicate that there is still significant room for improvement in its implementation and optimization. Our work underscored the ubiquity of at least 1 interventional cardiologists, 1 cardiac surgeon and 1 non-interventional cardiologist. However, the inclusion of other pivotal members, such anaesthetists, radiologists and patients, emphasizes the breadth and depth that a well-structured HT can bring to patient care. In line with the findings of our recent survey [7], most studies were found not to conform with recent guidelines, with the majority of studies not conforming to the recommended composition that includes a clinical cardiologist, interventional cardiologist, cardiac surgeon, imaging specialist and cardiovascular anaesthesiologist.

One of the cornerstones of the HT approach is its systematic deliberation of cases. While the frequency of meetings varied across studies, the significance of regular and comprehensive interactions was evident. Further insights from a study by Imran Hamid *et al.* [7] revealed that over 90% of respondents from various European institutions reported the presence of an HT. The study highlighted significant variability in the real-world practice of HTs across Europe. Fifty-five percent of these respondents held weekly HT meetings, with 73% being conducted face-to-face. This frequency and mode of interaction underscore the value of direct, regular communication in optimizing patient care.

Surprisingly, our review revealed that a majority of studies did not specify the use of structured templates. The importance of structured templates was evident in the findings of Imran Hamid *et al.* [7], where marked variability in documentation processes was observed. This omission might indicate an underutilization or underreporting of these essential tools. The use of structured templates, such as custom-made electronic forms or specific proformas, can enhance clarity, ensure consistency and streamline deliberations.

The integration of risk scores, such as SYNTAX and STS-PROM [28–30], further exemplifies the systematic nature of the HT approach. These scores provide objective data, aiding the team in assessing the complexity of coronary artery disease or predicting the risks associated with particular surgical procedures. Surprisingly, Imran Hamid *et al.* [7] noted that 67% of respondents did not calculate the SYNTAX score for coronary artery disease patients, highlighting potential gaps in systematic assessments. Their usage aligns with the ethos of the HT approach—evidence-based, comprehensive and patient-centric care. While it is acknowledged that different regions, such as the USA and Europe, have established preferences for certain risk scores—STS-PROM in the USA and EuroSCORE II in Europe—this diversity should not be viewed as a barrier to improving patient care. Instead, it provides an opportunity for cross-regional learning and the potential to integrate multiple risk scores into a more flexible and comprehensive framework.

This diversity reflects the unique healthcare environments in which these scores are used, but it does not preclude future efforts to harmonize risk stratification methods across regions. By adopting a flexible approach tailored to institutional needs, while remaining open to the benefits of alternative scoring systems, the HT model can continue to evolve and improve outcomes for patients. Ultimately, the goal should be to optimize the use of risk scores based on the best available evidence, ensuring that each institution employs the tools most appropriate for their patient population while fostering continuous improvement and consistency in decision-making across different settings (Figure 3).

### The role of multidisciplinary heart teams in improving patient outcomes

The findings from the reviewed studies underscore the critical role of HTs in improving patient outcomes for CAD, mitral and aortic valve diseases. One of the cardinal attributes of HTs is their intricate and thorough patient evaluation mechanism. For instance, Domingues *et al.* [9] discovered that additional investigations were solicited for a significant 35% of patients. When juxtaposed with the findings of Abdulrahman *et al.* [26], which showcased the efficacy and favourable outcomes of HT decisions in complex coronary cases, it becomes evident that such a meticulous evaluation paves the way for optimal patient care.

The collective wisdom of the HTs also fosters an adaptive and dynamic treatment strategy. Tsang *et al.* [23] illuminated this facet, noting that the HT’s collective decision-making process deviated from the initial treatment plan for nearly a third (30.3%) of aortic stenosis patients. Such adaptability not only showcases the team’s commitment to customizing care but also their agility in pivoting based on evolving patient data.

The efficacy of medical interventions is ultimately measured by their clinical outcomes, and the role of HTs is crucial in this regard. Evidence from Porterie *et al.* [20] underscores this point, demonstrating a significant decrease in in-hospital mortality for patients managed by an HT. Complementing this, research from Heuts *et al.* [10] and Jimenez *et al.* [11] further corroborates the practical advantages of the HT model, showcasing its contribution to improved treatment decisions and patient outcomes.

### Comparison between dedicated mitral valve heart team and general heart team

Reflecting on the data, it is noteworthy to observe that patients managed by a dedicated mitral valve HT exhibit superior outcomes compared to those managed by a general HT. In the research by Sardari Nia *et al.* [13], it was discernible that the patients under the dedicated HT’s care had better 5-year survival probabilities and lower adjusted relative risks of mortality.

The research demonstrates a statistically significant improvement in patient outcomes for those treated by a dedicated mitral valve HT, with a 5-year survival rate of 0.74, compared to 0.70 for patients treated by a general HT [95% confidence interval (CI) 0.66–0.74; *P* = 0.040]. A crucial finding is the adjusted relative risk of mortality, which was 29% lower for patients referred to the dedicated HT compared to the general HT (hazard ratio 0.71, 95% CI 0.54–0.95; *P* = 0.019). This comparative analysis unveils the significant advantage of a dedicated HT in managing mitral valve diseases. It suggests that a more focused and specialized team, possessing specific expertise and understanding of a particular disease entity, can yield better results in patient management.

Specialization improves communication and decision-making, focusing on the most effective interventions for better patient outcomes. This raises the question of whether specialized teams for other heart diseases could enhance care. However, the risk of fragmented care and increased costs from over-specialization suggests a balanced approach, combining specialized and generalist teams, is vital for patient-focused care.

### How to provide structure to the heart team?

Having a well-defined structure, complete with designated roles and responsibilities for each team member, creates a framework within which the team operates. This is crucial as it eliminates ambiguity, enhances coordination and ensures that every aspect of patient care is adequately addressed.

- Defined roles and responsibilities: ensures clarity, reduces ambiguity and promotes coordination.
- Robust communication channels: essential for ensuring effective collaboration and timely information sharing among team members.
- Decision-making protocols: decisions should be systematic, based on evidence, clinical guidelines and patient preferences. Avoid reducing decisions to mere opinions.
- Evaluation protocols: implement methods to integrate available evidence and assess treatment risks and benefits.
- Patient-centric approach: prioritize patient preferences and values in the decision-making process.
- Leverage technology: use shared electronic health records for better coordination. Incorporate decision support tools to align with the latest evidence and guidelines.

### Limitations of the field and the study

While dedicated HTs show promise in improving patient outcomes, several limitations should be acknowledged. The available evidence is largely based on single-centre, retrospective studies with small patient populations, which limits the ability to detect significant outcome differences. Additionally, the structure and function of HTs vary widely across institutions and countries. These differences are often inherent to the healthcare context, reflecting variations in resources, team composition and decision-making processes. Such variability is a natural consequence of differing healthcare systems and local practices, which affects how HTs operate and influences their efficacy in managing complex cardiovascular cases.

Moreover, there are no randomized controlled trials directly comparing dedicated HTs to general HTs, further complicating efforts to draw definitive conclusions about their relative effectiveness. The diversity of heart diseases, patient characteristics and treatment options across studies also makes it challenging to attribute improvements solely to the HT model.

Another important limitation is the lack of standardized metrics for evaluating clinical outcomes and the impact of HTs. The absence of such metrics makes cross-study comparisons difficult, introducing inconsistencies in outcome reporting. Additionally, potential delays in decision-making, biases in team recommendations and the possibility of bypassing thorough evaluations in routine cases highlight the need for more systematic approaches. Standardized protocols and quality audits would help address these issues, enabling HTs to deliver more consistent and effective patient care, while recognizing that some degree of variability is unavoidable due to the specific contexts in which these teams operate.

## CONCLUSION

The review highlights the HTs crucial role in cardiovascular care, pointing out significant variations in their makeup and operation. Inspired by oncology's success, HTs offer a model for collaborative patient management. The impact of HTs varies but shows positive trends, especially in mitral valve interventions with better repair rates and survival, aortic valve strategies tailored for better outcomes and nuanced coronary disease management. Despite these successes, challenges such as variability in HT structure—often inherent to the differing healthcare contexts across institutions and countries—and the lack of conclusive evidence from randomized controlled trials call for greater standardization and further study.

While some degree of variability is unavoidable due to the differences in resources, team composition and healthcare systems, the development of standardized protocols and outcome metrics could help optimize HT implementation and improve the comparability of results across studies. The balance between specialized and comprehensive care remains critical. As the healthcare field moves towards more collaborative, integrated and patient-focused care, there is a growing need for a model that combines specialized expertise with a comprehensive, standardized approach to maximize patient outcomes.

## Data Availability

The data that support the findings of this study are available from the corresponding author (P.SN), upon reasonable request.

## ABBREVIATIONS

ACC: American College of Cardiology
ACCF: American College of Cardiology Foundation
AHA: American Heart Association
CABG: Coronary artery bypass grafting
CAD: Coronary artery disease
CI: Confidence interval
EACTS: European Association for Cardio-Thoracic Surgery
ESC: European Society of Cardiology
HT: Heart team
NYHA: New York Heart Association
PCI: Percutaneous coronary intervention
TAVI: Transcatheter aortic valve implantation
VHD: Valvular heart disease
